# Anomalous X-ray diffraction on hybrid perovskite thin films: results and challenges

**DOI:** 10.1107/S1600576725009951

**Published:** 2025-11-28

**Authors:** Lena Merten, Paul Zimmermann, Niels Scheffczyk, Ekaterina Kneschaurek, Valentin Munteanu, Alina Weiss, Azat Khadiev, Alexander Hinderhofer, Frank Schreiber

**Affiliations:** a Institute of Applied Physics, University of Tübingen, Auf der Morgenstelle 10, 72076 Tübingen, Germany; b Deutsches Elektronen-Synchrotron DESY Notkestrasse 85 22607 Hamburg Germany; Universität Duisburg-Essen, Germany

**Keywords:** lead halide perovskites, anomalous X-ray diffraction, thin films, hybrid materials

## Abstract

Anomalous X-ray diffraction has been applied for structure determination in both biological and inorganic systems. The ability to create elemental contrast by varying the X-ray energy around the absorption edge of a specific element makes it a viable tool to probe and locate specific elements within a crystal structure, providing valuable complementary information to standard X-ray diffraction. Considering the large amount of research on hybrid organic–inorganic perovskites for photovoltaic applications, it is surprising that anomalous diffraction experiments on these materials are rather under-explored. In recent years, hybrid lead halide perovskite materials have evolved as a promising material class for next-generation photovoltaic devices, as well as further applications. X-ray diffraction methods, including grazing-incidence and *in situ* techniques, are routinely implemented for structural investigations. This work presents anomalous diffraction studies on each component of hybrid metal halide perovskites, *i.e.* metal cation, halide anion and central cation, providing an overview of the opportunities and challenges associated with applying anomalous X-ray diffraction to hybrid metal halide perovskites. The results are consistent with model calculations on (pseudo-)cubic perovskite structures.

## Abstract 2

Anomalous diffraction analysis of hybrid perovskite thin films enables quantification of components in mixed compositions, as well as prospective investigation of superlattice ordering and spatial component distribution.

## 1. Introduction

An anomalous scattering and diffraction experiment exploits the resonant behaviour of X-rays close to atomic absorption edges. X-rays are generally scattered by the electrons within a material that can be induced to oscillate. When the energy of the impinging X-rays matches the resonance energy of one of the inner-shell electrons, it can induce resonant excitation to a bound state, which can enhance dispersion effects near absorption edges (Materlik & Sparks, 1994 ▸; Hodeau *et al.*, 2001 ▸; Fuoss *et al.*, 1981 ▸; Shevchik, 1977 ▸). This causes variations in the intensity of Bragg reflections, which contain information on the location of the anomalous scatterers in a crystal structure, their partial structure and the occupation of atomic sites (Többens *et al.*, 2016 ▸; Ravel *et al.*, 2002 ▸; Collins *et al.*, 2007 ▸; Seshadri *et al.*, 1997 ▸; Nishihata *et al.*, 2002 ▸; Yamawaki *et al.*, 2001 ▸; Tsukimura *et al.*, 1990 ▸). Furthermore, the dispersion effects are sensitive to the chemical state and electronic environment at the probed atom site (Akao *et al.*, 2003 ▸; Wilkinson *et al.*, 1995 ▸; Grenier *et al.*, 2004 ▸), which is also employed in the related technique of resonant elastic X-ray scattering (REXS). Altering the resonant scattering factors by varying the X-ray energy can be used to adjust the contrast between different kinds of atoms in an alloy to investigate mixing or ordering behaviours via the partial structure of one or several components (Kemik *et al.*, 2011 ▸; Collins *et al.*, 2007 ▸; Collins *et al.*, 2015 ▸; Többens *et al.*, 2020 ▸; Nishihata *et al.*, 2002 ▸).

With its ability to create element-specific contrast, anomalous diffraction can provide answers to questions to which the ‘normal’ X-ray diffraction is oblivious. First and foremost, the presence of a certain element in a distinct crystallographic position can be determined by characteristic variations in peak intensities, which depend on the position of the element in question within the unit cell. A quantitative investigation can be performed, where the magnitude of the anomalous effect is expected to correlate with the proportion of the desired element within the crystal structure (Collins *et al.*, 2015 ▸).

For structures where nonequivalent positions within a unit cell can be occupied by different types of atoms, their ordering behaviour can be inferred by anomalous diffraction, as has been demonstrated before on the example of kesterite types of structures (Többens *et al.*, 2020 ▸) or Heusler alloys (Collins *et al.*, 2007 ▸). If similar crystallographic positions within a crystal structure can be occupied by different kinds of atoms, the mixing behaviour is expected to influence the anomalous effect due to a diverging interference pattern between the diffracted intensities from the anomalously scattering element.

**Figure 1:**
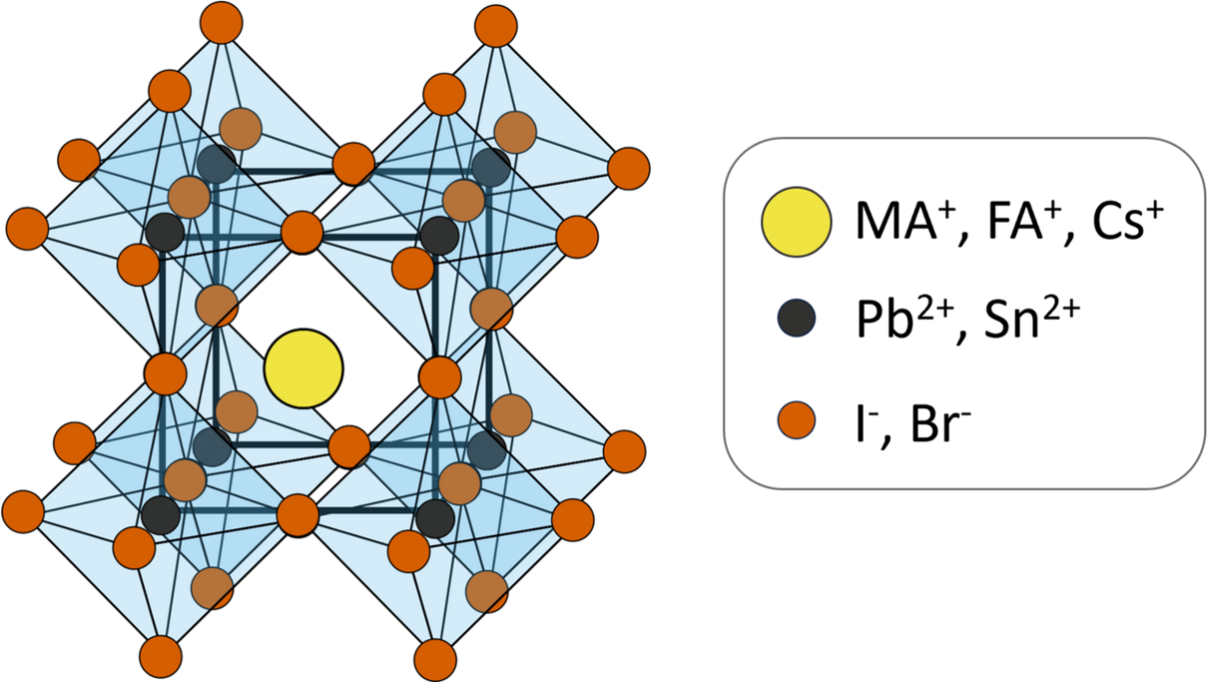
Schematic depiction of the perovskite structure with a list of frequently applied components. MA^+^ is methylammonium and FA^+^ is formamidinium. The cubic unit cell is indicated by a black outline.

Hybrid organic–inorganic perovskite materials can contain a wide range of elements, from lighter atoms such as carbon or nitrogen up to heavy metals like lead. Perovskite structures generally comprise three different types of ions, as shown schematically in Fig. 1 ▸, and are usually described by an *ABX*_3_ formula. In the specific case of the hybrid materials discussed here, *A* is either a small organic cation like methylammonium (MA^+^) or formamidinium (FA^+^), or in some cases a monovalent inorganic cation such as caesium. *B* describes a divalent metal cation, mostly lead or in rarer cases tin. Lastly, *X* stands for a halide anion, which is generally iodide, bromide or mixtures thereof. The structures and properties of hybrid perovskites are well described in the literature (Stoumpos *et al.*, 2013 ▸; Saliba *et al.*, 2018 ▸; Green *et al.*, 2014 ▸; Stoumpos & Kanatzidis, 2015 ▸; Greco *et al.*, 2018 ▸).

**Figure 2:**
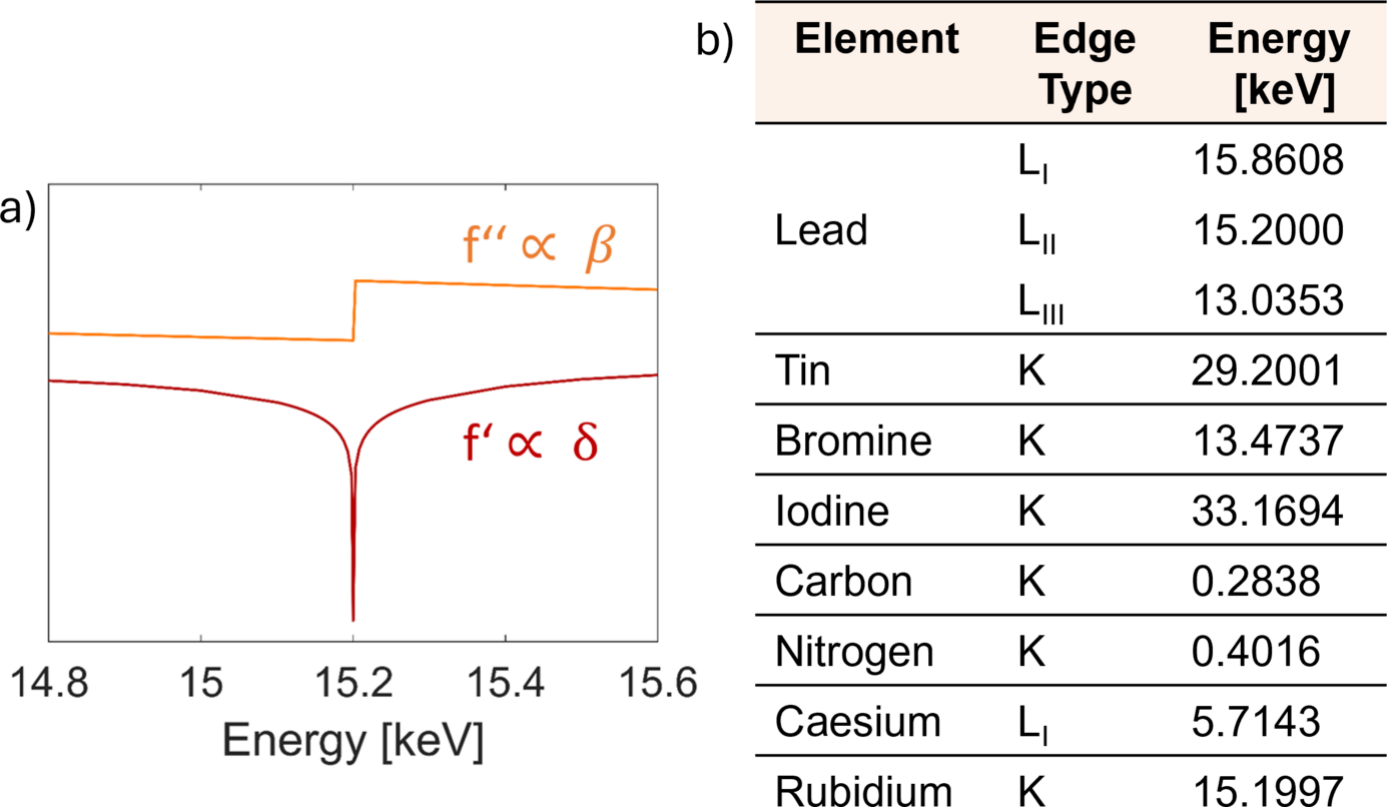
(*a*) Correction factors *f*′ and *f*′′ for the atomic form factor of lead close to the *L*_II_ absorption edge, as an illustration of the general behaviour around absorption edges. Units in electrons per atom. (*b*) Absorption edges in the X-ray regime, which can be employed for anomalous diffraction, for different commonly applied elements in hybrid perovskite compositions. All data taken from tabulated values (Chantler *et al.*, 1995 ▸; Merrit, 2023 ▸).

The wide range of atomic masses of the elements contained in hybrid perovskite structures leads to their absorption edge energies, which could possibly be utilized for anomalous diffraction experiments, spanning a wide energy range as well. For the lighter elements contained in the organic cations, these energies are below 1 keV, which is in the soft X-ray regime, requiring dedicated beamlines and experiments in vacuum to eliminate air absorption of the low-energy X-rays (Bishop *et al.*, 2024 ▸; Jarrige *et al.*, 2018 ▸; Jiao *et al.*, 2017 ▸; Carpenter *et al.*, 2015 ▸; Araki *et al.*, 2006 ▸). The heavier elements commonly contained in perovskite structures possess absorption edges between 10 and 30 keV [Fig. 2 ▸(*b*)], which is a range frequently employed for X-ray diffraction experiments and thus can be covered by many beamlines.

For inorganic perovskite materials, anomalous diffraction experiments investigating structural disorder (Petkov *et al.*, 2010 ▸; Richter *et al.*, 2018 ▸), superlattices (Kemik *et al.*, 2011 ▸) or charge ordering states (Akao *et al.*, 2003 ▸) have been widely reported.

Despite the opportunities offered by anomalous diffraction, to date the authors are not aware of extensive reports on anomalous diffraction experiments on hybrid lead halide perovskites. This is surprising considering the enormous amount of research dedicated to those materials. Thus, this work presents and evaluates measurements of anomalous diffraction on hybrid perovskites with different compositions to assess their prospects and challenges.

## 2. Background of the technique

The atomic form factor *f*, which quantifies the scattering ability of a certain type of atom, becomes a complex number close to the resonance energies of a specific element, *i.e.* absorption edges. Energy-dependent correction factors have to be applied, such that the atomic form factor can be written as
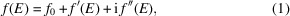
where *f*_0_ is the energy-independent ‘normal’ form factor due to elastic Thomson scattering, while *f*′ and *f*′′ are the resonant terms, which depend on beam energy and only possess non-negligible values close to absorption edges (Hodeau *et al.*, 2001 ▸; Shevchik, 1977 ▸). These are related to the macroscopic absorption coefficient μ and thus refractive index *n* of a material via the dispersion δ and absorption β terms: 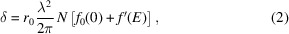

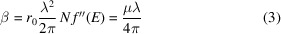
and 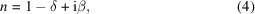
where λ is the X-ray wavelength, *r*_0_ the electron radius and *N* the sum over all atoms/ions within the unit cell of the probed material. The attenuation coefficient, μ, is experimentally accessible and can be used to determine the anomalous scattering factors *f*′′ and *f*′ via the Kramers–Kronig relations (Materlik & Sparks, 1994 ▸; Kronig, 1926 ▸).

Near an absorption edge, these resonant terms undergo strong variations, resulting in a sharp jump in *f*′′ and a dip in *f*′, as depicted in Fig. 2 ▸(*a*). These intensity variations can lead to phase changes in the structure factor. The structure factor determines the scattering intensity by taking into account the types and positions of atoms within the unit cell. It is described by 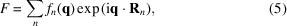
where the sum is executed over all atoms within the unit cell and **R**_*n*_ is their position within the unit cell. The final diffracted intensity *I* is proportional to the square of the structure factor, *I* ∝ |*F*|^2^. Thus, variations in the structure factor result in variations in the diffraction intensities as a function of beam energy (Hodeau *et al.*, 2001 ▸; Fuoss *et al.*, 1981 ▸). Depending on the position of the resonant scattering atom within the unit cell of a crystal structure and its contributions to the different scattering planes, this creates distinct intensity behaviours for each Bragg peak, sometimes even causing forbidden reflections to occur (Eichhorn *et al.*, 1988 ▸; Templeton & Templeton, 1986 ▸).

Generally, all elements with an atomic number *Z* ≥ 20 can be used for anomalous scattering employing hard X-rays (Ealick, 2000 ▸; Hodeau *et al.*, 2001 ▸); the absorption edges of their *K* or *L* transitions occur at energies between 4 and 40 keV, which is a typical energy range for X-ray diffraction experiments (Simon *et al.*, 1997 ▸). For an anomalous diffraction experiment, several diffraction patterns are recorded at different energies close to the absorption edge of the element in question within an energy span of ∼1 keV to track the intensity behaviour of the Bragg signals over the resonance edge. To track intensities successfully over several measurements, monitoring of the incoming beam flux, control of systematic errors and a sufficient counting time to increase the signal-to-noise ratio are vital, since the variations in signal intensity are usually much smaller than the total diffraction intensities of the Bragg peaks (Shevchik, 1977 ▸). A high energy resolution of about Δ*E*/*E* ≃ 10^−4^ and energy stability of the X-ray beam are required when performing several measurements around an absorption edge, as well as information on the detector efficiencies as a function of energy for absolute calibration.

The experimental data are then compared with models by simulating their expected energy-dependent diffraction patterns. For these calculations, the exact values of the anomalous scattering factors are required. These enter both the structure factor calculations and the crystallographic parameters of the material, including the atomic positions within the unit cell, geometric contributions, and factors for polarization and Lorentz correction (Buerger, 1940 ▸). Experimental parameters, such as radiation polarization, scattering angle 2θ and the azimuth of the crystal, which can influence reflection intensities, all require consideration, as do fluorescence effects that can create a large background intensity near absorption edges. This can be done by optimizing the setup by employing an energy-sensitive detector or simultaneous background measurements, or by subtracting tabulated values for background effects.

## 3. Experimental details

### 3.1. Sample preparation

For the precursor solution for CsPbBr_3_, lead bromide (PbBr_2_, ultra dry, 99.999% purity, Alpha Aesar) and CsBr (ultra dry, 99.999% trace metal basis, Sigma–Aldrich) were dissolved in anhydrous dimethyl sulfoxide (DMSO, ≥99.9%, Sigma–Aldrich) at a concentration of 0.33 mol l^−1^ and a molar ratio of CsBr:PbBr_2_ of 2.75:1 by stirring overnight at 60°C and 1000 rpm. The solution was filtered through a 0.2 µm poly(tetrafluoroethylene) filter before thin-film deposition. The precursor ink (150 µl) was deposited onto a plasma-cleaned glass substrate and spin-coated at 4000 rpm for 120 s. The thin films were annealed at 100°C on a hotplate for 20 min.

Hot-pressing was applied to the annealed thin films at 100 bar and 150°C for 30 min. For CsSnBr_3_ thin films, CsBr and SnBr_2_ were dissolved in a mixture of *N*,*N*-dimethyl­formamide (DMF, anhydrous, 99.8%, Sigma–Aldrich) and DMSO (1:4 *v*/*v*) at a concentration of 0.33 mol l^−1^. Additionally, 5% (0.0165 mol l^−1^) SnF_2_ was added to the precursor solution, which was stirred overnight at 60°C. The precursor ink (100 µl) was deposited onto a plasma-cleaned glass substrate and spin-coated at 2000 rpm for 120 s; 30 s before the end of spin-coating, nitrogen gas quenching was applied, which is reported elsewhere (Brinkmann *et al.*, 2019 ▸). The thin films were annealed at 60°C on a hotplate for 10 min. Hot-pressing was applied to the annealed thin films at up to 200 bar and 200°C.

**Figure 3:**
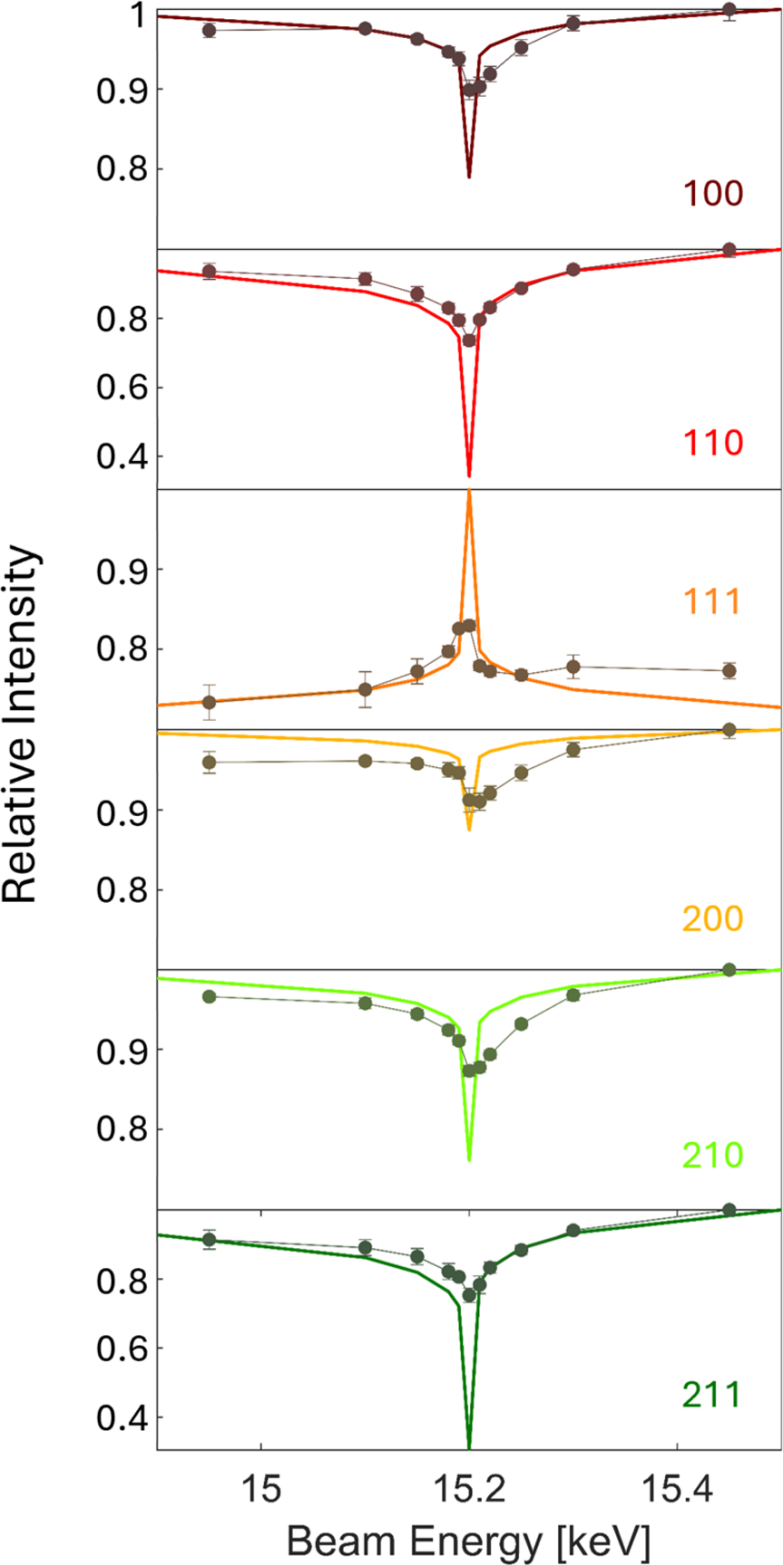
Normalized peak intensities of selected Bragg peaks as a function of beam energy around the Pb *L*_II_ edge for an FA_0.85_MA_0.15_PbI_3_ thin film. Thick solid lines are simulated data (Gratia *et al.*, 2017 ▸; Merrit, 2023 ▸) and darker markers with thin lines are experimental data. Coloured numbers in the graphs indicate the Miller indices of the respective diffraction peaks.

Mixed-cation perovskite thin films, as well as the sample featured in Fig. 3, were fabricated using a single-step deposition method from the precursor solution containing FAI (1.0 *M*), PbI_2_ (1.1 *M*), MABr (0.2 *M*) and PbBr_2_ (0.2 *M*) in anhydrous DMF (99.8%)/DMSO (99.7%) (4:1 *v*/*v*). For the triple-cation composition, 5% CsI (1.5 *M* in DMSO, ultra-dry, 99.998%) was added to the precursor solution. To prepare the quadruple composition, 5% RbI (1.5 *M* in DMF/DMSO, 4:1 volume ratio) was added to the triple-cation composition. The precursor solutions were spin-coated in a two-step process at 1000 and 6000 rpm for 10 and 30 s, respectively. During the second step, 100 µl of chlorobenzene (99.8%) was added dropwise onto the spinning substrate 10 s prior to the end of the process. This was followed by annealing the films at 100°C for 45 min.

Mixed halide perovskite thin films with MAPbBr_*x*_I_1−*x*_ composition were fabricated via the gas-quenching technique, similarly to previously reported samples (Brinkmann *et al.*, 2019 ▸; Merten *et al.*, 2024 ▸). Precursor solutions were prepared from PbI_2_ (AnhydroBeads, 99.999%, Sigma–Aldrich), PbBr_2_ (99.999%, Sigma–Aldrich), MAI (anhydrous, 99.99%, Dye­namo) and MABr (anhydrous, 99.99%, Dyenamo) in a 4:1 (*v*/*v*) mixture of DMF (biotechnology grade, ≥99.9%, Sigma–Aldrich) and DMSO (anhydrous, ≥99.9%, Sigma–Aldrich) dissolved in a nitrogen atmosphere with a concentration of 1.4 *M* using stoichiometric precursor amounts. Thin-film deposition took place in a specifically designed deposition system (Kneschaurek *et al.*, 2023 ▸). The films were spin-coated at 3000 rpm for 150 s; after 15 s, a nitrogen flow was directed at the sample to establish gas quenching. The thin films were annealed at 100°C for 4–10 min.

Additional samples were fabricated by the lead acetate route (Sun *et al.*, 2017 ▸). MAI and MABr (≥99.99%, Great Cell Solar) were dissolved in a drybox (relative humidity ≤ 0.4%) in DMF (anhydrous, Sigma–Aldrich) to a 42 wt% concentration, and then hypophosphorous acid (HPA, 50:50 vol.% in water, Alfa Aesar; 1.7 µl per 63 µmol methyl­ammonium halide) was added. Appropriate amounts of methylammonium halide solution (halide/lead 3:1) were either added directly to lead acetate trihydrate (≥99%, Sigma–Aldrich) or mixed by volume beforehand to form mixtures of halides. The final solutions were spin-coated onto bare or ITO-coated glass (soda lime glass, pre-cleaned with acetone, propan-2-ol and oxygen plasma) at 2000 rpm for 60 s in a DMF-rich atmosphere. Directly at the end of the spin-coating, a compressed dry air flow was directed at the as-deposited thin film for 20 s. After being dried for 5 min in dry air, the samples were annealed for 5 min at 100°C in the same environment.

### 3.2. Anomalous diffraction measurements

Anomalous diffraction experiments were conducted on beamline P23 at DESY (Hamburg, Germany), which provides an energy resolution of Δ*E*/*E* = 1.3 × 10^−4^ and an accessible energy range of 5–35 keV. The samples were kept in an evacuated environment under a Kapton dome during the experiment, while the beam path between the sample and the detector was in air. The detector distances were varied between 200 and 300 mm, while both an X-Spectrum Lambda 750K and a Dectris PILATUS3 X 1M detector were used to record two-dimensional diffraction patterns to include the diffraction intensities from differently oriented crystallites. Energy calibration was done using a gold foil at the Au *L*_II_ and *L*_III_ edges (13.734 and 11.919 keV, respectively). The energy precision of the X-ray beam was 0.5 eV and the beam size was (horizontal × vertical) 300 × 100 µm. For each experiment, several separate data sets were acquired using several samples of the same composition and scanning each sample several times. In this way the measuring statistics could be improved, so that a meaningful powder average was achieved.

The lead *L*_II_ edge, tin *K* edge, bromine *K* edge, caesium *L*_I_ edge and rubidium *K* edge as listed in Fig. 2 ▸(*b*) were chosen for anomalous measurements (Merrit, 2023 ▸). Measurements were performed at 15 energies around the chosen edge energy: ±5, ±10, ±20, ±50, ±100, ±250 and ±500 keV, and at the edge energy itself.

Since the samples were polycrystalline thin films, the measurements were performed in reflection geometry with small incidence angles in the range of 1–5°, depending on sample composition.

The changing detector efficiency with X-ray energy was accounted for directly on the beamline by performing calibration measurements with a non-anomalously scattering test sample (LaB_6_) within the energy ranges that were also applied for measurements. That way, the efficiency of the detector could be recorded as a function of energy and directly applied to the measured data: *I*_2_ = *I*_raw_/Eff_Det_, where Eff_Det_ is the energy-dependent detector efficiency, *I*_raw_ the measured intensity and *I*_2_ the efficiency-corrected intensity.

An avalanche photodiode detector was used to monitor the incoming beam intensity, recording the incident beam scattering from the Kapton foil of the exit opening of the X-rays. The energy efficiency of the monitor counts could be determined in the same way as for the detector. Both detector and monitor efficiencies generally only have a small linear effect and are thus not the most critical for data shape. During measurements, monitor counts were recorded continuously and then the data were normalized by the monitor counts before further processing to account for input intensity variations: *I*_3_ = *I*_2_/mon, with the monitor counts mon and monitor-corrected intensity *I*_3_.

The data were converted from angular space to *q* space by MATLAB scripts, resulting in energy-independent peak positions. Intensity correction for the transformation was achieved using the factor 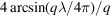. Afterwards, the 2D data were numerically integrated into 1D curves.

Intensity variations due to fluorescence effects were accounted for by tracking the measured intensity outside of diffraction peaks and subtracting it as a baseline correction: *I*_bgcorr_ = *I*_int_ − *I*_bg_, with *I*_int_ the 1D integrated intensity curve, *I*_bg_ the background intensity and *I*_bgcorr_ the background-corrected intensity.

From the background-corrected intensity curves, peak intensities were integrated by fitting a Gaussian function and tracked for different Bragg peaks as a function of energy. Radial profiles (*i.e.* azimuthally integrated intensities over a 90° azimuthal angle) were used for randomly oriented samples to account for the intensity of the full diffraction ring. For a polycrystalline thin film, the azimuthal averaging mitigates the effects of different crystal azimuths on the diffraction intensity. For more spot-like peaks, separate integration wedges were applied, using geometric correction factors according to scattering probabilities for the azimuthal angle.

X-ray absorption in the surrounding air was accounted for by a linear model using tabulated values (Chantler *et al.*, 1995 ▸). For the transmission factor, 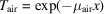 was used, where μ_air_(*E*) is the linear absorption coefficient of air and *x* is the path length between the sample and detector for X-rays in air. The distance in air between the source and sample was kept minimal. Note that air absorption also has to be considered when determining detector efficiencies.

Similarly, for the X-ray absorption of the sample itself, the linear absorption coefficient μ(*E*) was used, which was obtained by interpolation of tabulated values (Chantler *et al.*, 1995 ▸), assuming stoichiometric composition: 
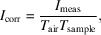
where *I*_meas_ is the measured efficiency-corrected intensity and 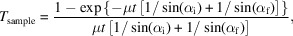
with *t* the film thickness and α_i,f_ the incidence and exit angles, respectively. The absorption edge position of the correction curve, obtained for the pure element from the tabulated values, was adjusted to the experimentally observed one using the fluorescent background.

Any possible resonant Raman effects are expected to be very weak and are thus treated as part of the background.

Energy-dependent diffraction patterns were simulated using the simulation mode of *FullProf.2k* (Arcelus *et al.*, 2024 ▸) in combination with *MEADmaker* (Többens *et al.*, 2020 ▸) on the basis of crystal structures extracted from the literature (Stoumpos *et al.*, 2013 ▸; López *et al.*, 2019 ▸; Evans *et al.*, 2018 ▸; Gratia *et al.*, 2017 ▸; Marstrander & Moller, 1966 ▸). For the mixtures, crystal parameters and occupation factors were adjusted according to stoichiometry. The energy-dependent *f*′ and *f*′′ factors were interpolated from tabulated data (Merrit, 2023 ▸).

## 4. Results and discussion

### 4.1. Anomalous diffraction of the metal cation

Lead is generally the heaviest element in hybrid perovskite compositions, and thus it has the strongest X-ray scattering power of the components. It is also expected to produce a distinct anomalous effect. The presence and position of the lead cation within a perovskite structure are undebated, so measurements of anomalous diffraction on the lead *L*_II_ absorption edge can serve as a proof of principle for the possibility of anomalous diffraction experiments on hybrid perovskite thin films. We chose the *L*_II_ edge because its simpler and sharper peak allowed the use of a narrower energy scan range than for the *L*_III_ edge.

Fig. 3 ▸ shows the measured and simulated intensity variations for anomalous diffraction experiments along the Pb edge on FA_0.85_MA_0.15_PbI_3_ perovskite thin films. Distinct maxima and minima are observed for the energy-dependent intensities of the different diffraction signals, depending on the phase of the contributions of lead to the respective scattering plane. The qualitative behaviours of calculated and experimentally measured peak intensities as a function of energy coincide well, indicating the possibility of detecting lead at its dedicated position in a perovskite structure. The main difference between the simulated curves (coloured lines in Fig. 3 ▸) and the experimental data (darker markers in Fig. 3 ▸) is the relative change in total intensity, represented in the figure as the sharpness of the peak. This is probably connected to the chosen beam energy values in the experiment, causing the experimental data not to follow the calculated curve exactly, due to fewer data points being measured than were used in the calculation. High X-ray absorption by lead might also contribute to the effect being slightly smaller than expected. Additionally, the measured values are subject to the energy resolution of the instrument which can reduce the apparent effect of energy broadening. In the presented measurement, this is not considered to be a limiting factor, since the energy sampling step size is considerably larger than the energy resolution of the beamline (10 eV minimum sampling step size versus an energy resolution of 1.95 eV at 15 keV).

**Figure 4:**
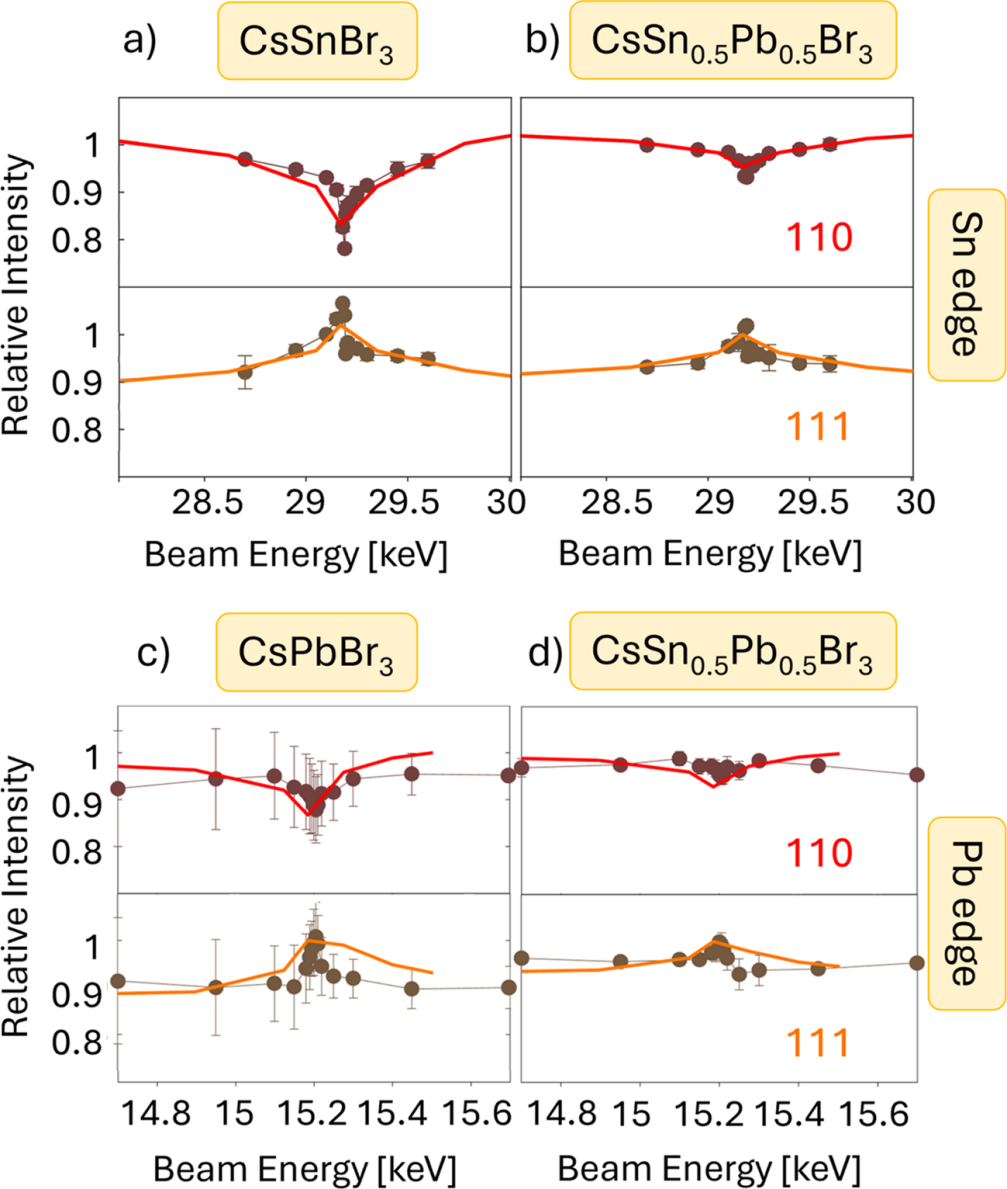
Normalized peak intensities of selected Bragg peaks as a function of beam energy around the Sn *K* edge for (*a*) CsSnBr_3_ and (*b*) CsSn_0.5_Pb_0.5_Br_3_ thin films, and around the Pb *L*_II_ edge for (*c*) CsPbBr_3_ and (*d*) CsSn_0.5_Pb_0.5_Br_3_ thin films. Thick coloured lines are simulated data (Marstrander & Moller, 1966 ▸; Merrit, 2023 ▸), assuming a random mixture of tin and lead. Darker markers with thin lines are experimental data. Coloured numbers in the graphs indicate the Miller indices of the respective diffraction peaks. Corresponding data on more diffraction peaks for both edges can be found in the supporting information in Fig. S1, while reciprocal-space maps for corresponding compositions are shown in Fig. S2.

The introduction of tin cations replacing lead in some metal cation positions within the perovskite structure has been proposed as a means to reduce lead toxicity in hybrid perovskite materials (Fan *et al.*, 2020 ▸). Although this route can be considered only an interim stage in the current search for a less toxic alternative, since tin possesses significant toxicity as well (Babayigit *et al.*, 2016 ▸), different methods have been proposed to introduce tin into existing lead halide perovskite structures. For several of them, a proof of inclusion onto the desired crystallographic position is required. This purpose can be served by anomalous diffraction on the Sn *K* edge. The corresponding experimental anomalous intensity variations in Fig. 4 ▸(*a*) for pure-tin CsSnBr_3_ perovskite thin films are consistent with the calculated data. For mixed lead–tin mater­ials, simulations predict qualitatively similar intensity behaviour to that of the pure-tin composition, but with reduced amplitudes in accordance with the reduced tin composition [Fig. 4 ▸(*b*)]. This is further reflected in the experimental data, which indicate successful inclusion of tin in the desired concentration on the same crystallographic position as lead.

In this case, fewer data points were used to simulate the curves than in Fig. 3 ▸, which were chosen as close as possible to the actually measured data points to make the calculated and measured intensity curves also quantitatively comparable. It can be seen in Fig. 4 ▸ that the curves agree well. Analysing the extent of the anomalous effect, *i.e.* the change in intensity of the diffraction signal, leads to the ability for quantitative determination of the tin concentration within a margin of about 10%. Thus, anomalous diffraction can be successfully applied to prove the presence of heavy elements on distinct crystallographic positions.

Quantitative statements on the concentration may be made, but care must be taken with the experimental conditions. For the determination of exact concentrations, precise values for the diffracted intensities are required. Several effects can influence and modify the measured values, including sample and setup absorption and possible re-emission, environmental scattering, detector efficiencies, input intensity variations, and more. Although some of these parameters can be accounted for by a parallel measurement of the corresponding phenomena during either the experiment or calculations, for the most precise measurement all of these factors require exact control. This is challenging under real-world conditions, since several of these effects such as absorption and re-emission mutually influence each other. Careful correction for sample absorption is required in order to quantify the results. For details of how these challenges were handled in the measurements presented here, an extensive description can be found in the *Experimental details*[Sec sec3] section.

Ultimately, anomalous diffraction offers an alternative method to determine the composition of certain perovskite materials within a thin film after fabrication. This holds significant potential for materials characterization and quality control.

### 4.2. Anomalous diffraction of the halide

**Figure 5:**
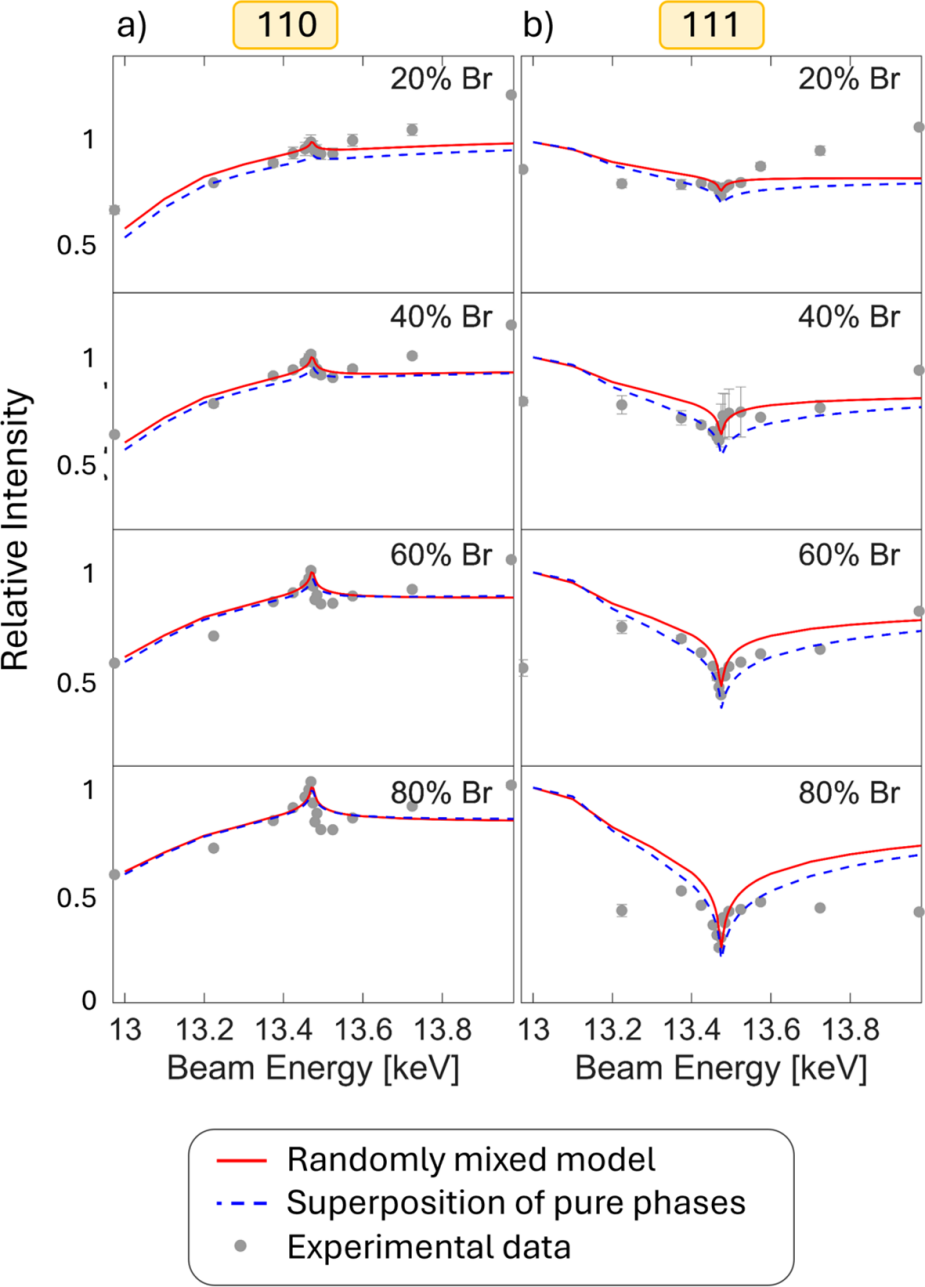
Normalized peak intensities of selected Bragg peaks as a function of beam energy around the Br *K* edge for MAPbBr_*x*_I_1−*x*_ compositions. Calculated data (Stoumpos *et al.*, 2013 ▸; López *et al.*, 2019 ▸; Evans *et al.*, 2018 ▸; Merrit, 2023 ▸) are displayed for a randomly mixed model and a superposition of mixed phases, together with experimental data for compositions of *x* = 0.2, 0.4, 0.6 and 0.8. (*a*) Intensity of the 110 diffraction peak and (*b*) intensity of the 111 diffraction peak. Calculated intensities for further diffraction peaks for the mixed model are shown in Fig. S3 in the supporting information, while reciprocal-space maps for corresponding compositions can be found in Fig. S4.

Apart from metal cation blends, mixed halides are commonly applied for perovskite structures (Noh *et al.*, 2013 ▸; Chen *et al.*, 2022 ▸; Knight & Herz, 2020 ▸). The mixing of halides in different ratios was investigated by employing the bromide *K* edge in MAPb(Br_*x*_I_1−*x*_)_3_ thin films, which is displayed in Fig. 5 ▸ together with simulated data. A distinct dependence of the magnitude of the anomalous effect on the concentration of bromide is visible. Similarly to what was shown above for the lead–tin mixtures, quantification of the halide composition can be achieved by anomalous diffraction data.

The simulations in Fig. 5 ▸ in one case assume a random mixture of iodide and bromide throughout the material, both occupying the halide position within the perovskite structure in a randomly intermixed arrangement. This is compared with a second case, a model of two separate bromide- and iodide-based phases. The expected differences between the two models are rather small, since the overall behaviour of the intensities does not change, *i.e.* there is no peak inversion or disappearance. The models only differ slightly in the peak shape. Since reliably measuring such subtle differences in peak shape is not achieved with the present experimental data, we cannot reach a definite conclusion on the mixing behaviour in this case. Note that the different slopes between experimental and calculated data at energies below the Br edge are probably related to the relatively close Pb *L*_III_ edge at 13 keV.

**Figure 6:**
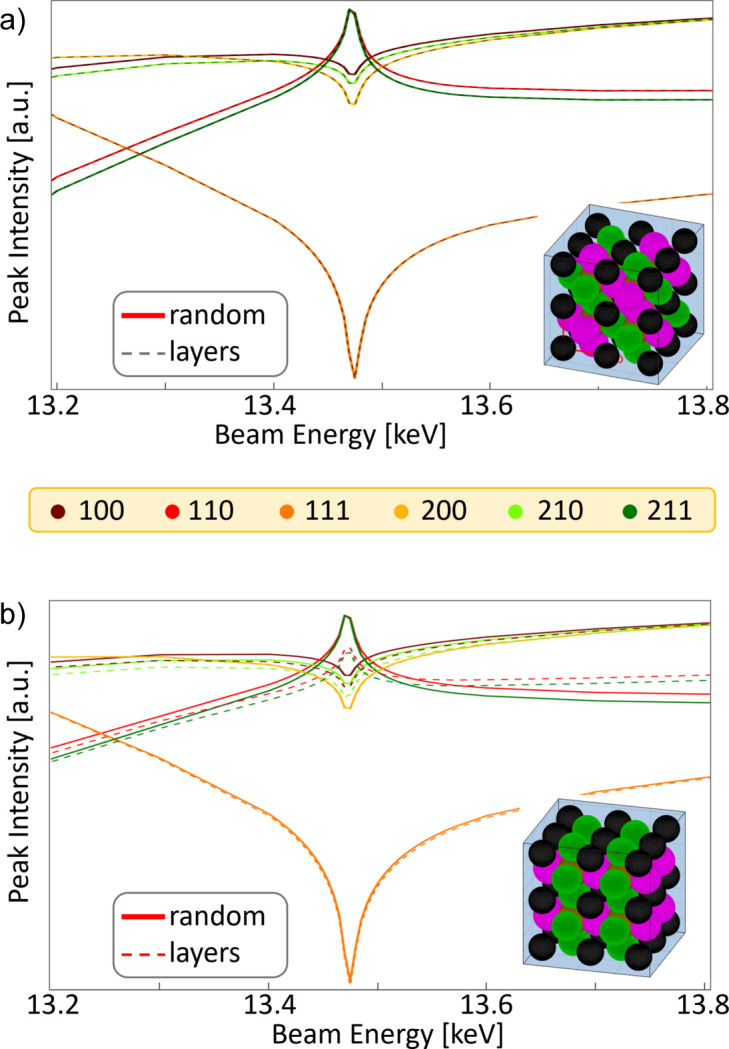
Simulated intensities of various Bragg peaks as a function of beam energy around the Br *K* edge for MAPbBr_*x*_I_1−*x*_ compositions, comparing a randomly mixed model with layered arrangements of (*a*) diagonal layers, *x* = 0.5, and (*b*) vertical layers, *x* = 0.33. The insets visualize the respective layered models. Green spheres indicate iodide, pink spheres bromide, black spheres the organic cation and brown spheres lead. The numbers in the legend indicate the Miller indices of the respective diffraction peaks.

An ordering of the halide anions in different types of arrangements, as shown in Fig. 6 ▸, can be imagined as well. These ordered arrangements are expected to create distinct interference patterns between the ordered halides, modifying the intensity behaviour of the anomalous effects. The calculations of the relative intensity variations for the different mixing models reveal that the expected effects do not alter the qualitative behaviour of the peak intensities, but cause only small deviations compared with the overall intensity trends. Thus, similar considerations apply for the ordering behaviour and for the mixing behaviour. The signal-to-noise ratio needs to be optimized to detect the tiny variations between the curves that are calculated theoretically. These tiny variations between the anomalous diffraction effects for different mixing patterns are considered to be caused by the fact that all potential positions for halides in a lead halide perovskite are equivalent, and thus each contributes to the diffraction intensity in a similar way and with the same scattering phase. For materials containing non-equivalent positions within a unit cell that can be occupied by two different kinds of ions, the differences for distinct mixing patterns are expected to be larger and more reliably measurable (Wilkinson *et al.*, 1995 ▸).

### 4.3. Anomalous diffraction of the central cation

Besides the metal cation and the halide anion, the third component in hybrid halide perovskite structures is the central cation, which is often a small molecular cation. However, since these are composed solely of light elements such as carbon or nitrogen, anomalous diffraction around their absorption edges would have to be measured at very low X-ray energies, and in a different setup to the heavier elements.

In hybrid perovskite compositions, it has become a common technique to replace or mix the organic cations with caesium or rubidium cations (Saliba *et al.*, 2016*a* ▸; Saliba *et al.*, 2016*b* ▸; Park *et al.*, 2017 ▸; Wang *et al.*, 2018 ▸; Merten *et al.*, 2021 ▸). Due to their larger atomic masses, the absorption edges are expected at higher energies as listed in Fig. 2 ▸(*b*). Preliminary test measurements of anomalous diffraction at the caesium and rubidium absorption edges on Cs_0.2_Rb_0.2_(FA_0.85_MA_0.15_)_0.6_PbI_3_ perovskite thin films can be found in the supporting information (Fig. S6 and Supplementary Note 1).

For caesium, the *L*_I_ edge at 5.71 keV is still relatively low, but nonetheless within the scope of many conventional diffraction beamlines. Additional considerations apply for such measurements; the low X-ray energy leads to increased air absorption, generally lower transmitted beam intensity and reduced efficiency of the detector in this energy range, causing a considerable reduction in the detected intensity and thus lowering the signal-to-noise ratio. Another challenge is linked to measuring anomalous diffraction of rubidium cations in perovskite compositions where lead is present, due to the overlap of the absorption edges. Therefore, more specialized lead-free compositions would be better suited to this experiment.

## 5. Conclusions

Compared with standard X-ray diffraction at X-ray energies far from absorption edges, anomalous diffraction is a relatively infrequently used technique, despite its potential to answer questions that cannot be resolved with standard diffraction. Setups and measurements for anomalous diffraction need to be carefully optimized for each specific experiment. Since the power of anomalous diffraction has been demonstrated on other material types, more effort is required to establish it as a technique to answer suitable questions on hybrid perovskite structures.

In this paper it has been shown that anomalous diffraction can be applied to detect certain elements in sufficient concentrations occupying distinct crystallographic positions. For small amounts this is challenging, but such challenges can be overcome with optimization of measurement techniques. To elucidate mixing behaviours, the perovskite structure with its relatively high symmetry, in which all positions for a certain species (or mixture thereof) are equivalent, poses a challenge. Instead, structures with reduced symmetry, such as layered structures, are expected to be more easily accessible.

A quantification of the amounts of specific components such as tin, lead or bromide has been shown to be feasible within a viable range by exploitation of the magnitude of the intensity changes caused by the anomalous effect. This provides new insights for materials characterization and quality control. Pursuing the optimization of anomalous diffraction experiments on hybrid perovskite materials further can therefore provide a valuable tool for materials characterization and development.

## 6. Related literature

For further literature related to the supporting information, see Hu *et al.* (2017 ▸), Kubicki *et al.* (2017 ▸), Mitchell (1959 ▸), Trots & Myagkota (2008 ▸), Uchida *et al.* (2018 ▸) and Zhang *et al.* (2017 ▸).

## Supplementary Material

Supporting information file. DOI: 10.1107/S1600576725009951/uz5028sup1.pdf

## Data Availability

Data will be available upon request from the corresponding authors.
