# Supplementary material for: Anomalous X-ray diffraction on hybrid perovskite thin films: results and challenges
Source: J Appl Crystallogr. 2025 Nov 28;58(Pt 6):2125–33. doi: 10.1107/S1600576725009951 (PMC12810543; doi:10.1107/S1600576725009951)
Supplement: Supplementary file 1 [file j-58-02125-sup1.pdf]

JOURNAL OF
APPLIED
CRYSTALLOGRAPHY

Volume 58 (2025)

Supporting information for article:

Anomalous X-ray diffraction on hybrid perovskite thin films: results and challenges

Lena Merten, Paul Zimmermann, Niels Scheffczyk, Ekaterina Kneschaurek, Valentin Munteanu, Alina Weiss, Azat Khadiev, Alexander Hinderhofer and Frank Schreiber

Anomalous X-ray Diffraction on Hybrid Perovskite Thin Films: Results and Challenges

Lena Merten [a], Paul Zimmermann [a], Niels Scheffczyk [a], Ekaterina Kneschaurek [a], Valentin Munteanu [a], Alina Weiss [a], Azat Khadiev [b], Alexander Hinderhofer [a], and Frank Schreiber [a]

[a]Institute of Applied Physics, University of Tübingen, Auf der Morgenstelle 10, 72076 Tübingen, Germany

[b]Deutsches Elektronen-Synchrotron DESY, Notkestr. 85, 22607 Hamburg, Germany

Supporting Information

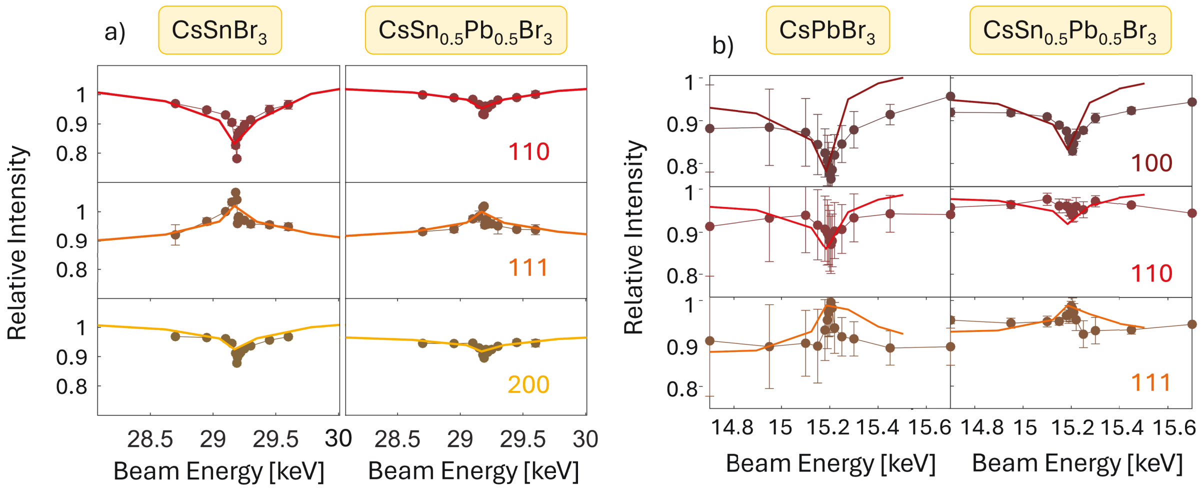

Figure S 1: Normalized peak intensities of selected Bragg peaks as a function of beam energy around (a) the Sn K-edge for $CsSnBr_3$ and $CsSn_{0.5}Pb_{0.5}Br_3$ thin films. (b) the Pb L_{II}-edge for $CsPbBr_3$ and $CsSn_{0.5}Pb_{0.5}Br_3$ thin films. Thick colored lines: simulated data (Marstrander & Moller, 1966; Merrit, 2023), assuming a random mixture of tin and lead. Darker markers with thin lines: experimental data. Colored numbers in the graphs indicate Miller indices of the respective diffraction peaks.

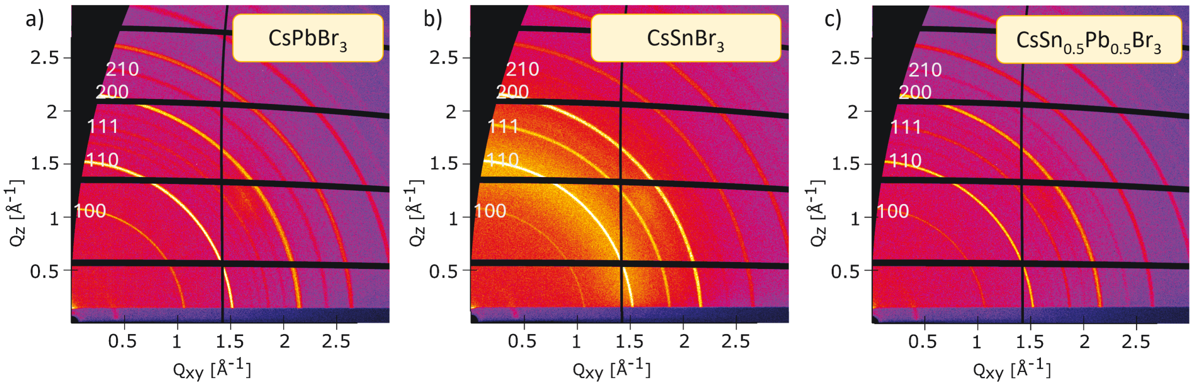

Figure S 2: Reciprocal space maps of Sn and Pb-based inorganic perovskite thin films at a beam energy of 15 keV and an incidence angle of 1°. (a) $CsPbBr_3$, (b) $CsSnBr_3$ and (c) $CsSn_{0.5}Pb_{0.5}Br_3$. White numbers are the miller indices of the respective diffraction signal.

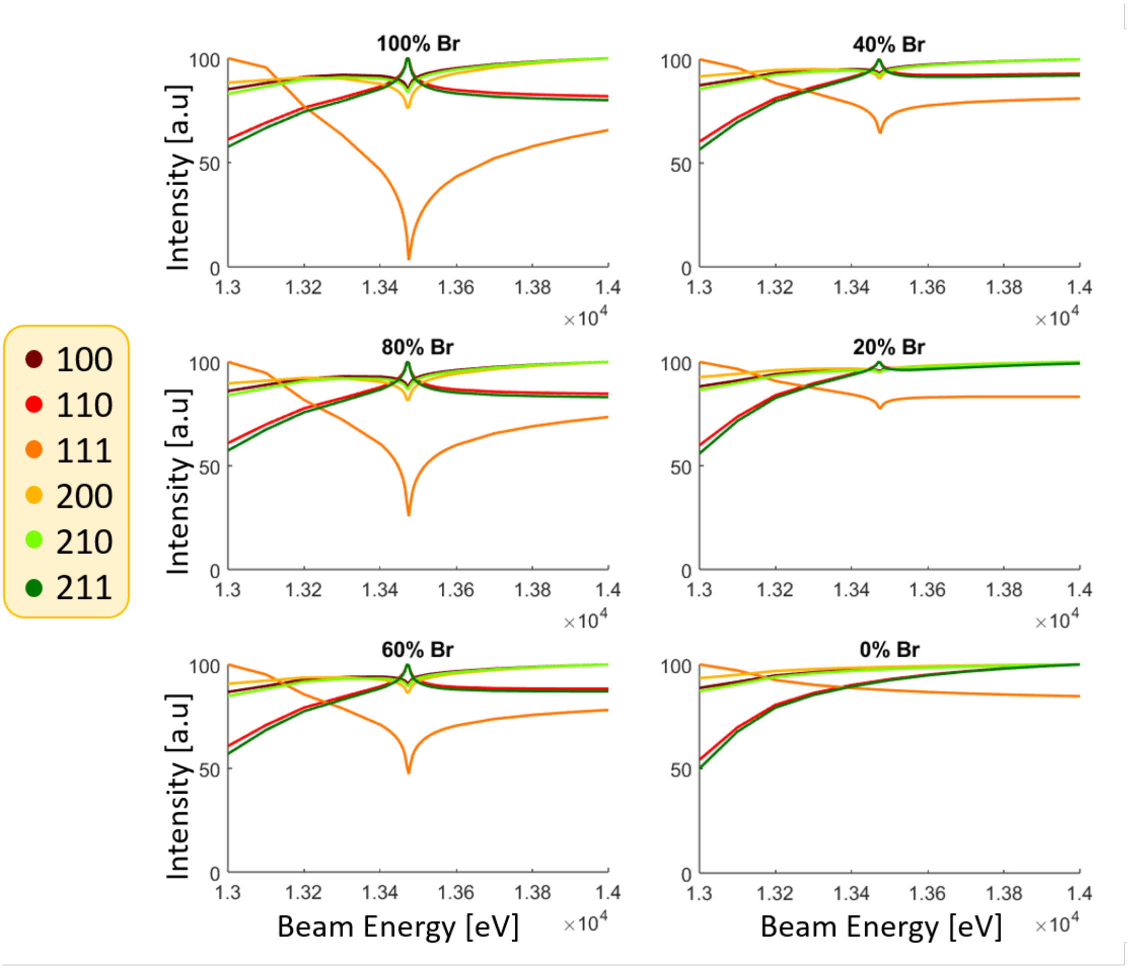

Figure S 3: Peak intensities of selected Bragg peaks as a function of beam energy around the bromide K-edge for $MAPbBr_xI_{1-x}$ compositions for different mixing ratios and a randomly mixed model for bromide and iodide. Numbers in the legend indicate Miller indices of the respective diffraction signals.

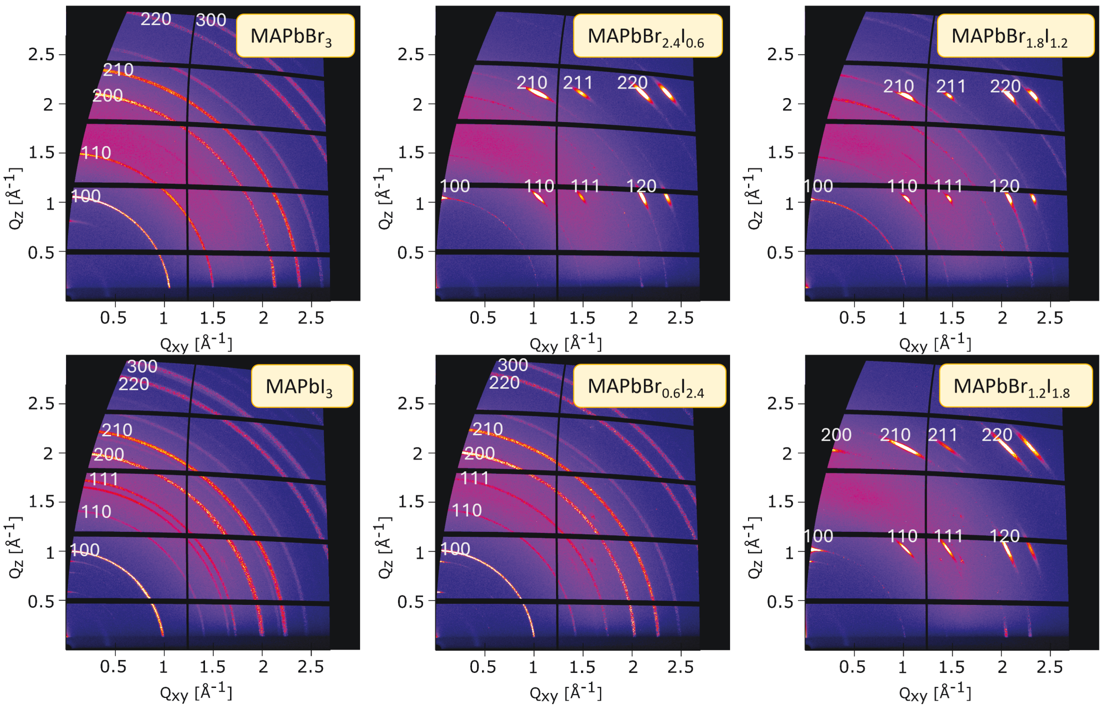

Figure S 4: Reciprocal space maps at an incidence angle of 1° and a beam energy of 13.974 keV for mixed halide $MAPbBr_xI_{1-x}$ compositions for different mixing ratios. White numbers are the miller indices of the respective diffraction signal.

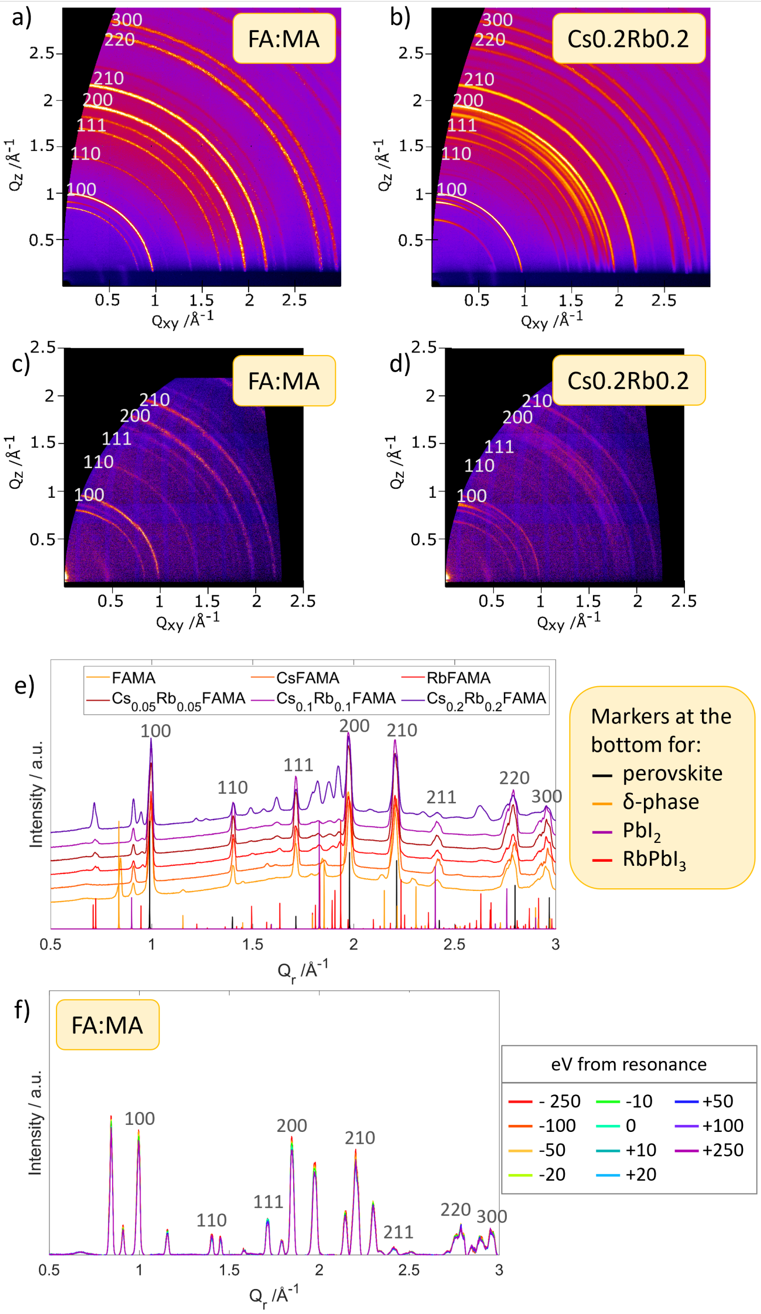

Figure S 5: Raw data and exemplary analysis pathway for evaluating anomalous diffraction experiments on $FA_{0.85}MA_{0.15}PbI_3$ thin films with rubidium and cesium cation addition. GIWAXS reciprocal space maps at (a), (b) E = 15.45 keV; (c), (d) E = 5.26 keV and $\alpha_i = 1°$. (e) Radial profiles at 15.45 keV with calculated peaks from literature (Gratia *et al.*, 2017; Trots & Myagkota, 2008; Mitchell, 1959), showing a mixture of phases. (f) Radial profiles of $FA_{0.85}MA_{0.15}PbI_3$ sample at different energies around the Pb L_{II} resonance edge at 15.2 keV, showing intensity variations. Miller indices of the diffraction signals are given by the white/grey labels.

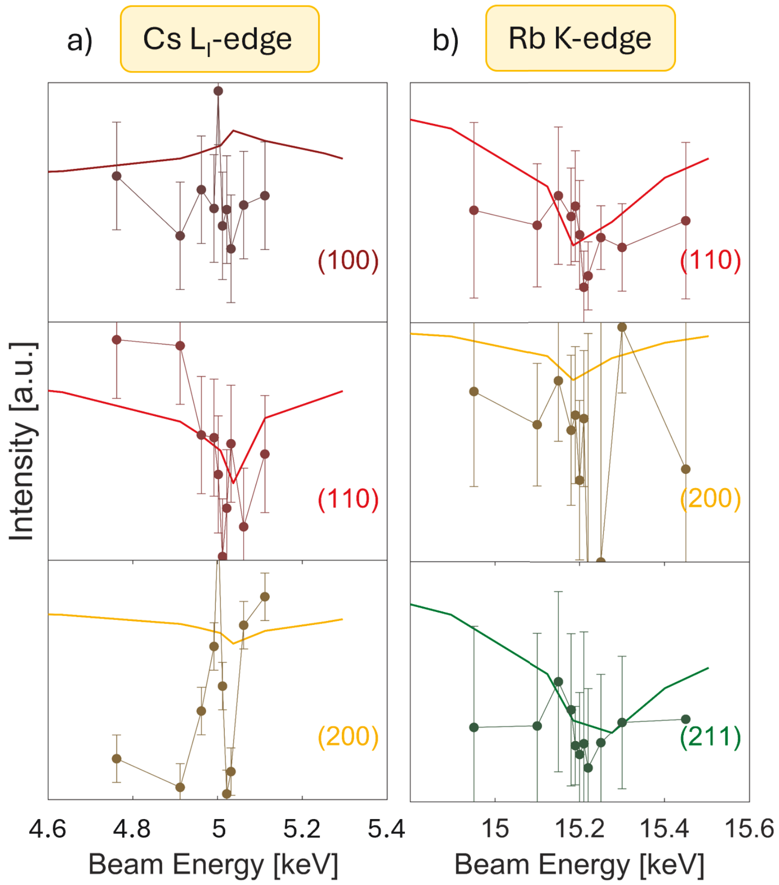

Figure S6: Normalized peak intensities of selected Bragg peaks as a function of beam energy around (a) the cesium L_I-edge and (b) the rubidium K-edge for $Cs_{0.2}Rb_{0.2}(FA_{0.85}MA_{0.15})_{0.6}PbI_3$ thin films. Thick colored lines: simulated data (Gratia *et al.*, 2017; Merrit, 2023), darker markers with thin lines: experimental data. Colored numbers indicate Miller indices of the respective diffraction peaks. Raw data, i.e. reciprocal space maps and radial profiles of mixed cation samples are shown in Figure S5.

Supplementary Note 1

Mixed cation perovskite thin films employing the widely applied mixture of $MA_{0.15}FA_{0.85}PbI_3$ with 20 % of cesium iodide inclusion were subjected to anomalous diffraction experiments around the cesium L_I-edge, which is displayed in Figure S6 (a). As is visible there, no clear anomalous effect can be observed in the data. Though some intensity variations between the measurements at different beam energies can be observed, no clear trend corresponding to the expected curves can be found. This is likely caused by different factors, including the possibility that cesium is not included into the perovskite structure in the desired crystallographic position, but it might likewise be possible that the included amount of 20 % is too low to be seen clearly. Additionally, the low X-ray energy leads to increased air absorption, generally lower transmitted beam intensity and reduced efficiency of the detector in this energy range, causing a considerable reduction of the detected intensity and thus lowering the signal-to-noise ratio that might limit the signal, which likely explains the intensity variations observed between measurements. Thus, experiments with improved parameters as well as higher amounts of cesium are required to possibly see the anomalous effect on cesium. This was however not the goal of the presented experiment, where the objective was to determine whether cesium has been incorporated into the mixed FA:MA perovskite composition in the given concentration. This can not yet be answered with this kind of experiment. As it has been shown for the mixed halide samples in the main text (Figure 5), in the sample with 20 % of bromide the anomalous effect was just barely visible, thus there might exist a detection limit for the relative amount of an anomalously scattering element in a certain position, depending on the experimental setup and parameters.

For the slightly heavier rubidium cation, the energies of the absorption edges are much higher than for cesium, so it is expected to be easier to measure and face less of the challenges caused by the low beam energy. However, as listed in Figure 2 (b) in the main manuscript, the rubidium K-edge, which would be in a convenient energy range for anomalous diffraction experiments, practically coincides with the lead L_{II}-edge. This lead edge is well-observed in anomalous diffraction experiments, as presented above. In theory, a superposition between the effects of lead and rubidium on the intensity behavior is expected. Thus, a possible route of resolving this issue could be the subtraction of the anomalous diffraction intensities of a reference $MA_{0.15}FA_{0.85}PbI_3$ sample without rubidium to eliminate the lead effect. This results in the data presented in Figure S6 (b), which shows no clear anomalous effect of the rubidium cation, but rather disorganized intensity variation. That might relate to this method of simple superposition and subtraction not being applicable or, as for cesium, to the low amount of 20 % of rubidium that was applied in the current samples. Thus, these small amounts of alkali cations are likely not detectable by anomalous diffraction experiments. Lastly, as many reports state rubidium as immiscible into the FA:MA-based perovskite phase (Zhang *et al.*, 2017; Kubicki *et al.*, 2017; Hu *et al.*, 2017; Uchida *et al.*, 2018), it might simply not be present in the desired crystallographic position and thus not be detectable in the diffraction intensities of the perovskite phase at all. However, this conclusion cannot be drawn from the present data, since, similarly to the cesium cation, the expected amounts of rubidium would be low

and might not be detectable.

Thus, while in mixed compositions larger amounts of components ($\geq 20\,\%$ as shown for bromide above) in dedicated crystallographic positions can be successfully detected by anomalous diffraction experiments, this is likely the detection limit with the current setup, especially when facing additional complications such as low beam energy or overlapping absorption edges as in the presented experiments.

References

Gratia, P., Zimmermann, I., Schouwink, P., Yum, J.-H., Audinot, J.-N., Sivula, K., Wirtz, T. & Nazeeruddin, M. K. (2017). *ACS Energy Lett.* **2**(12), 2686–2693.

Hu, Y., Aygüler, M. F., Petrus, M. L., Bein, T. & Docampo, P. (2017). *ACS Energy Lett.* **2**(10), 2212–2218.

Kubicki, D. J., Prochowicz, D., Hofstetter, A., Zakeeruddin, S. M., Grätzel, M. & Emsley, L. (2017). *J. Am. Chem. Soc.* **139**(40), 14173–14180.

Marstrander, A. & Moller, C. K. (1966). *Mat. Fys . Medd. Dan. Vid. Selsk.* **35**(5), 1–12.

Merrit, E. A. (2023). *Biomolecular Center, University of Washington; available at http://skuld.bmsc.washington.edu; accessed*, A**pr.**, 19.

Mitchell, R. S. (1959). *Z. Kristallogr. – Cryst. Mater.* **111**(1-6), 372–384.

Trots, D. & Myagkota, S. (2008). *J. Phys. Chem. Solids*, **69**(10), 2520–2526.

Uchida, R., Binet, S., Arora, N., Jacopin, G., Alotaibi, M. H., Taubert, A., Zakeeruddin, S. M., Dar, M. I. & Grätzel, M. (2018). *Small*, **14**(36), 1802033.

Zhang, M., Yun, J. S., Ma, Q., Zheng, J., Lau, C. F. J., Deng, X., Kim, J., Kim, D., Seidel, J., Green, M. A., Huang, S. & Ho-Baillie, A. W. Y. (2017). *ACS Energy Lett.* **2**(2), 438–444.
